# Draft Genome Sequence of *Fusarium fujikuroi*, a Fungus Adapted to the Fuel Environment

**DOI:** 10.1128/genomeA.01499-17

**Published:** 2018-01-18

**Authors:** Osman Radwan, Thusitha S. Gunasekera, Oscar N. Ruiz

**Affiliations:** a Environmental Microbiology Group, University of Dayton Research Institute, Dayton, Ohio, USA; b Fuels and Energy Branch, Aerospace Systems Directorate, Air Force Research Laboratory, Wright-Patterson AFB, Ohio, USA

## Abstract

*Fusarium fujikuroi* isolate FUS01 is highly adapted to grow in jet fuel with predicted genes involved in hydrocarbon catabolism and carbon assimilation. The draft genome size is estimated at 49 Mb containing 18,578 proteins with high similarity to that of *F. fujikuroi* isolate B14.

## GENOME ANNOUNCEMENT

Although the genomes of several fuel-adapted bacteria have been reported (1–3), far fewer reports have described the genomes of fuel-adapted fungi (4). The ability of the filamentous fungus *Fusarium fujikuroi* isolate FUS01 to adapt and grow in fuel-containing environments enthused us to sequence its genome.

A fungal isolate was recovered from a sample of Jet A fuel and identified as *F. fujikuroi* isolate FUS01 based on morphological characteristics and high similarity (99%) of the 18S rRNA gene to that of *F. fujikuroi*. Using a whole-genome shotgun approach, TruSeq paired-end libraries were generated and sequenced on a HiSeq 2000 platform, resulting in 49,249,613 paired-end reads with a read length of 100 bp (~4.92 Gb). The raw sequences were trimmed using Trimmomatic (5), and reads shorter than 40 bp were discarded. The sequence reads were *de novo* assembled with SPAdes software (6). The resulting 49,057,912-bp (100× sequence coverage) draft assembly comprises 881 contigs greater than 500 bp and has an *N*_50_ of 838,232 bp and an *L*_50_ of 16 contigs. The G+C content of the assembled genome is 47.34%, which is less than the 48.3% G+C content of *F. fujikuroi* isolate B14 (GenBank accession number ANFV00000000), the causal agent of the bakanae disease of rice (7). CEGMA (8) was then run on the draft assembly and identified 243 out of 248 ultraconserved eukaryotic genes in *F. fujikuroi* isolate FUS01 (97.98%). After masking repetitive sequences (2.80%) using the RepeatMasker program (9), the masked genome was used for gene prediction by AUGUSTUS version 2.5.5 (10) with an option set for *F. graminearum* parameters, resulting in 18,578 protein-coding genes.

Based on a BLASTp search against the UniRef90 and UniProt databases, significant matches (E value, 1E^−5^) were identified for 16,572 proteins; 15,427 hits were derived from *F. fujikuroi* isolate B14, while tRNAscan-SE (11) identified 299 tRNAs. A total of 967 fungal enzymes involved in carbohydrate metabolism and assimilation were identified (E value, 1E^−4^) using the Carbohydrate-Active Enzyme (CAZy) database (12). These enzymes include polysaccharide lyases (*n =* 25), glycosyl transferases (*n =* 118), glycoside hydrolases (*n =* 396), carbohydrate esterases (*n =* 193), carbohydrate-binding modules (*n =* 106), and auxiliary activities (*n =* 129).

The KEGG database and BLASTp research identified important proteins related to biofilm formation (agglutinin-like proteins, *n =* 17) and efflux of toxic substances (efflux pumps, *n =* 74), which are well studied in bacteria (2), suggesting a common mechanism of hydrocarbon adaptation between bacteria and fungi. Similarly, proteins involved in carbon metabolism (*n =* 81) and degradation of aromatic compounds (*n =* 9), such as benzoate 4-monooxygenase and cyclohexanone monooxygenase, are found. In agreement with the capability of FUS01 to adapt and grow in fuel, we found genes involved in the oxidation of alkanes. These genes include the AlkB-related alkane hydroxylases CYP153 and CYP505 and the *n*-alkane-inducible cytochrome P-450 (13). Information from the current genome will facilitate an understanding of the mechanisms underlying fungal adaptation to, growth in, and degradation of hydrocarbon fuels.

### Accession number(s).

This whole-genome shotgun project has been deposited at DDBJ/ENA/GenBank under the accession number NCQQ00000000. The version described in this paper is the second version, NCQQ02000000.
